# Tuberculosis treatment outcome and predictors in northern Ethiopian prisons: a five-year retrospective analysis

**DOI:** 10.1186/s12890-018-0600-1

**Published:** 2018-02-20

**Authors:** Kelemework Adane, Mark Spigt, Geert-Jan Dinant

**Affiliations:** 1 Department of Medical Microbiology and Immunology, College of Health Sciences, Mekelle University, PO Box 1871, Mekelle, Ethiopia; 2 Department of Family Medicine, Maastricht University/CAPHRI School for Public Health and Primary Care, PO Box 616, Maastricht, the Netherlands; 3 General Practice Research Unit, Department of Community Medicine, UiT the Arctic University of Norway, PO Box 6050, Tromsø, Norway

**Keywords:** Tuberculosis, Treatment outcome, Prisons, Ethiopia

## Abstract

**Background:**

The prison situations are notorious for causing interruptions of tuberculosis (TB) treatment and occurrence of unfavorable outcomes. In Ethiopian prisons, though TB treatment programs exist, treatment outcome results and factors contributing to unsuccessful outcome are not well documented. In this study, we assessed the treatment outcome of TB cases and identified risk factors for unsuccessful outcome in northern Ethiopian prisons.

**Methods:**

A retrospective record review was conducted for all prisoners diagnosed with TB between September 2011 and August 2015. Outcome variables were defined following WHO guidelines.

**Results:**

Out of the 496 patients, 11.5% were cured, 68% completed treatment, 2.5% were lost to follow-up, 1.6% were with a treatment failure, 1.4% died, and 15% were transferred out. All transferred out or released prisoners were not appropriately linked to health facilities and might be lost to treatment follow-up. The overall treatment success rate (TSR) of the 5 years was 94% among the patients who were not transferred out. The odds of unsuccessful outcome were 4.68 times greater among re-treatment cases compared to the newly treated cases. The year of treatment was also associated with variations in TSR; those treated during the earlier year were more likely to have unsuccessful outcome. Sputum non-conversion at the second-month check-up was strongly associated with unsuccessful outcome among the smear-positive cases.

**Conclusions:**

The mean TSR of the prisoners in the study prisons was quite satisfactory when gauged against the target level set by the End TB Strategy. However, the lack of appropriate linkage and tracking systems for those prisoners transferred or released before their treatment completion would have a negative implication for the national TB control program as such patients might interrupt their treatment and develop drug-resistant TB. Being in a re-treatment regimen and sputum non-conversion at the second-month check-up were significantly associated with unsuccessful treatment outcome among the all forms of and smear-positive TB cases, respectively.

## Background

Globally, the burden of tuberculosis (TB) is higher in vulnerable populations such as prisoners and is reported to be up to 100 times higher than in the general population [1]. In Sub-Saharan African prisons, TB remains as one of the fastest growing epidemics [2, 3]. The high prevalence of human immunodeficiency virus (HIV) infection and the lack of well-organized TB diagnostic and treatment systems [2, 4] contribute to the disproportionate burden of TB in the Sub-Saharan African prisons.

Inadequate TB treatment will lead to the emergence of drug-resistant strains [5]. The prison situations are notorious for causing interruptions of TB treatment and occurrence of unfavorable outcomes [6]. In some prisons, up to 24% of the TB cases have been shown to harbor multidrug-resistant TB (MDR-TB) which makes TB control efforts very complicated [7]. In Russian and Brazilian prisons, 12% [8] and 8% [9] of the TB cases have been reported to default their treatment, respectively. In a Ugandan prison, 43% of the prisoners with TB had defaulted their treatment [10]. The TB treatment category, HIV co-infection, smoking, alcoholism, and a lack of family support have been indicated as factors affecting TB treatment success in prisons [8, 9, 11].

In Ethiopian prisons, TB treatment programs exist and are integrated within the national TB control program where the prison health staff provides treatment based on the national guidelines [12]. However, treatment outcome results and the potential factors for unsuccessful outcome are not well documented. According to a report in 2013 from the prison of North Gondar, the treatment success rate (TSR) of the prisoners ranged from 42 to 80% within the 10 years period [13]. However, this study was not comprehensive in that it did not assess the potential factors that might be affecting the treatment success. Evaluating the treatment outcome results and identifying the risk factors for the unsuccessful outcome will help to identify the gaps between the national TB treatment policy and practice in prisons and initiate evidence-based interventions. This study was designed to assess the treatment outcome of TB cases and identify risk factors for unsuccessful outcome in northern Ethiopian prisons.

## Methods

### Study setting and diagnostic criteria

This study was carried out in prisons of the Tigray Regional State, northern Ethiopia. Out of the nine prisons in Tigray, four prisons, located in the cities Alamata, Humera, Mekelle and Shire, were randomly selected and included in the study. Information regarding the TB diagnostic and treatment services in these settings have been described elsewhere [14]. In general, conditions of the four prisons were comparable where the prisons had only poorly equipped clinics staffed with diploma holding nurses. There were also no sputum microscopy, GeneXpert or drug susceptibility testing (DST) services in the clinics of the prisons and the TB diagnosis relied merely on a referral of prisoners to health facilities outside prisons [14]. The diagnosis was carried out at the referral sites using the direct smear microscopy and/or chest X-ray, and pathological investigation following the national guidelines [15]. Accordingly, a presumptive TB case with at least two initial sputum smear examinations positive for AFB (acid-fast bacilli) by direct smear microscopy or one sputum examination positive for AFB and having radiographic abnormalities consistent with active pulmonary TB is considered as a smear-positive TB (PTB+) case. A patient having symptoms suggestive of TB with at least three initial smear examinations negative for AFB on the direct microscopy and with radiological abnormalities consistent with pulmonary TB is defined as a smear-negative TB (PTB−) case. On the other hand, if a patient has TB involving organs other than lungs as proven by histopathological evidence from a biopsy or based on strong clinical evidence consistent with active extra-pulmonary TB (EPTB) he/she is categorized as having EPTB.

Prisoners diagnosed with TB were linked to the directly observed treatment short-course (DOTS) clinics of the nearby health facilities where they were registered and started the treatment according to the national guidelines [15]. Afterwards, the prison health personnel collected drugs weekly from such facilities and continued the treatment within prisons. The treatment is given based the patients’ treatment category. Newly diagnosed TB cases are provided with the 6 months treatment regimen where they take a combination of four drugs (rifampicin, isoniazid, pyrazinamide, and ethambutol) during the first 2 months of the intensive phase and continue with two of the drugs (rifampicin and isoniazid) for the remaining 4 months. On the other hand, the previously treated cases are treated for 8 months. This regimen consists of 8 weeks treatment with streptomycin, rifampicin, isoniazid, pyrazinamide, and ethambutol followed by 4 weeks treatment with rifampicin, isoniazid, pyrazinamide, and ethambutol during the intensive phase, followed by 5 months with rifampicin, isoniazid, and ethambutol. Bacteriological follow-up examinations (sputum smear checkups) are done at the end of the second, fifth, and sixth month of therapy for all new sputum-positive patients and at the end of the intensive phase of treatment (the third month), and at the end of the fifth and eighth months of treatment for the previously treated sputum smear-positive patients. The treatment follow-up data was regularly reported back to the health facilities for registration as the study prisons lacked standardized logbooks to register.

### Study design and data collection

This was a retrospective analysis in which the profile and treatment outcomes of all prisoners diagnosed with TB between September 2010 and August 2015 was retrieved from the TB treatment follow-up clinics of the four selected prisons. Patients’ information such as age, sex, the type of TB case, the treatment category, the date of treatment initiation and completion, weight at the time of treatment initiation, HIV status, and other related data were recorded by the prison nurses using a standardized recording format.

### Outcome definition

The referral sites and/or the DOTS centers use the standard national TB case definitions and treatment outcomes adopted by WHO [16]. The categories of outcome include: cured (a TB patient who was smear- or culture-positive at the beginning of the treatment but who became smear- or culture negative in the last month of treatment and on at least one previous occasion), treatment completed (finished the treatment with resolution of symptoms but without smear or culture result), treatment failure (remained or became smear-positive at the end of 5 months or later), lost to follow-up (missed treatment for at least eight consecutive weeks), transferred out (transferred to another site during the treatment), died (patients who died from any cause during the course of treatment). We further grouped the outcomes into successful treatment (sum of cured and treatment completed) and unsuccessful treatment which is the sum of treatment failure, loss to follow-up, and death as per the WHO standard definition [17].

### Data analysis

Data were entered using Epi Data entry version 3.1 software and analyzed using SPSS version 21. We compared prisoners with unsuccessful treatment outcome with those having successful outcome. Even though it was already one of the elements of the unsuccessful outcome, death was specifically considered as an additional outcome variable because we were interested to see its relationship with body weight at treatment initiation. Bivariate and multivariate logistic regression analyses were performed to examine the association of independent variables with our outcome variables. Covariates with *p*-values of ≤0.25 and collinearity matrix index of ≤0.7 in the bivariate analysis were considered for inclusion in the multivariate model. A *p*-value of ≤0.05 was considered to define a statistical significance.

## Results

### Socio-demographic and clinical characteristics

The baseline characteristics of TB patients (*n* = 496) are shown in Table 1. The majority (97%) was male, and the mean age was 30 years (range 15–18 years). Extra-pulmonary TB patients accounted for 43% of the TB cases; 30% of total cases had PTB−. Out of the 123 (25%) PTB+ cases, repeated smear was done for 119 (97%) at the end of the 2nd month of treatment and nine were still smear-positive. Eleven percent of the TB cases were HIV-infected; the co-infection rate being slightly higher among EPTB cases (13%). However, the difference was not statistically significant.

**Table 1:** Socio-demographic and clinical characteristics of the 496 patients with tuberculosis in northern Ethiopian prisons, 2011–2015

Characteristic	*n* (%)
Sex	
Male	480 (97)
Female	16 (3)
Age, years	
15–24	201 (40)
25–34	173 (35)
≥35	122 (25)
Weight at treatment initiation (Kg)	
<50	221 (45)
≥50	275 (55)
Form of TB	
PTB+	123 (25)
PTB−	150 (30)
EPTB	211 (43)
EPTB & PTB	12 (2)
Treatment category	
New case	481 (97)
Retreatment case	15 (3)
HIV status	
Negative	424 (85)
Positive	54 (11)
Unknown	18 (4)
Antiretroviral treatment (ART)^a^	
On ART at time/within in 3 months of TB treatment	46 (85)
Not on ART	3 (6)
Unknown	5 (9)
Use of co-trimoxazole prophylaxis^a^	
On co-trimoxazole at time/within 3 months of TB treatment	40 (74)
Not on co-trimoxazole	14 (26)
Site	
Alamata	73 (14)
Humera	163 (33)
Mekelle	183 (37)
Shire	77 (16)

^a^The denominator is HIV co-infected patients (*n* = 54); *HIV* human immunodeficiency virus, *TB* tuberculosis, *PTB+* smear-positive pulmonary tuberculosis, *PTB−* smear-negative pulmonary tuberculosis, *EPTP* extra-pulmonary tuberculosis

### Trend of the TB types

**Fig. 1:**
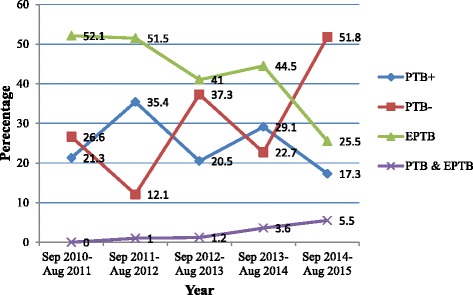
Trends of all forms of registered TB cases (*n* = 496) in the DOTs center of four northern Ethiopian prisons, 2010–2015. Aug: August, Sep: September; PTB+: smear-positive pulmonary tuberculosis; PTB−: smear-negative pulmonary tuberculosis; EPTP: extra-pulmonary tuberculosis

The 5-year trend of all the TB types is shown in Fig. 1. The number of PTB− cases showed variation across years steadily increasing in the recent year; more than half of the TB cases (51.8%) were PTB− between the September 2014 and August 2015. On the other hand, the number of EPTB cases roughly decreased over the 5 years.

### Treatment outcome and trends

**Fig. 2:**
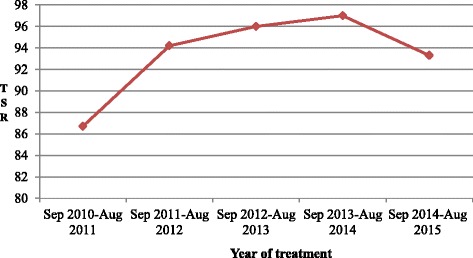
Trends of treatment success rate for all forms of the TB cases (*n* = 496) from four northern Ethiopian prisons, 2010 to 2015. Aug: August; Sep: September; TSR: Treatment Success Rate; TB: Tuberculosis

The mean treatment success rate (TSR) of the 5 years was 94% (395/422) among the patients who were not transferred out. As shown in Fig. 2, the TSR steadily increased from 87% during September 2010–August 2011 to 97% in September 2013–August 2014. Overall, 57 (11.5%) were cured, 338 (68%) had completed treatment, 12 (2.5%) were lost to treatment follow-up, 8 (1.6%) were with treatment failure, 7 (1.4%) died, and 74 (15%) were transferred out with outcome unknown. All the prisoners transferred to other prisons or released during the course of their TB treatment were not appropriately linked to health facilities.

### Treatment success rate and associated factors

The treatment success rate and associated predictors are shown in Table 2. After excluding the transferred out prisoners and dichotomizing the outcome variable, further analysis was done for the 422 cases. In the multivariable analysis, the odds of having unsuccessful outcome was significantly higher among the retreatment cases compared to the newly treated ones (AOR = 4.68; 95% CI = 1.02–21.4). Prisoners that were treated during September 2010–August 2011 were also more likely to have unsuccessful outcome, compared to those treated during September 2011–August 2012 (AOR = 0.28; 95% CI = 0.08–0.92), September 2012–August 2013 (AOR = 0.24; 95% CI = 0.06–0.98), and September 2013–August 2014 (AOR = 0.17; 95% CI = 0.05–0.67). Sputum non-conversion at the second-month check-up was strongly associated with unsuccessful outcome among the smear-positive cases. There was no association between unsuccessful outcome and HIV status, the weight at treatment initiation, the study site, and the form of the TB case. When we perform the analysis considering death as an outcome variable, the odds of dying among patients with a weight at initiation of <50 Kg was 8.4 times higher (OR = 8.39; 95% CI = 1.01–70.34) compared to the other group.

**Table 2:** Factors related to unsuccessful treatment outcome among northern Ethiopian prisoners with tuberculosis in the bivariate and multivariate logistic regression analysis (n = 422). AOR: adjusted odds ratio; COR: crude odds ratio; CI: confidence interval; ref = reference

Characteristic	Treatment outcome		COR (95% CI)	P-value	AOR (95% CI)	P-value
	Unsuccessful n (%)	Successful n (%)				
Sex						
Female	2 (14.3)	12 (85.7)	2.56 (0.54-12.04)	0.26	-	
Male	25 (6.2)	383 (93.8)	ref			
Age, years						
15-24	8 (4.6)	164 (95.4)	0.62 (0.23-1.71)	0.36	-	
25-34	11 (7.9)	129 (92.1)	0.86 (0.86-1.09)			
> 35	8 (7.3)	102 (92.7)	ref			
Weight at initiation (Kg)						
< 50	15 (8.4)	164 (91.6)	1.76 (0.83-3.86)		1.72 (0.75-3.98)	0.2
> 50	12 (4.9)	231 (95.1)	ref			
Form of TB^a^						
PTB+	8 (7.6)	97 (92.3)	1.22 (0.48-3.14)	0.48	-	
PTB -	8 (6.1)	124 (93.9)	0.96 (0.37-2.45)	0.38		
EPTP	11 (6.3)	163 (93.7)	ref			
Non-conversion at the 2nd month^b^						
Yes	2 (12.5)	4 (87.5)	9.1 (1.33-62.16)	0.02	-	
No	5 (5.2)	91 (94.8)				
Type of TB case						
Retreatment case	3 (25.0)	9 (75.0)	5.36 (1.36-21.11)	0.01	4.68 (1.02-21.4)	0.04
New case	24 (5.8)	386 (94.2)	ref			
HIV status^C^						
Positive	4 (8.5)	43 (91.5)	1.36 (0.45-4.14)	0.58	-	
Negative	23 (6.4)	338 (93.6)	ref			
Year						
Sep 2010-Aug 2011	10 (13.3)	65 (86.7)	ref			
Sep 2011-Aug 2012	5 (5.8)	81 (93.2)	0.41 (0.13-1.23)	0.11	0.28 (0.08-0.92)	0.04
Sep 2012-Aug 2013	3 (4.2)	69 (95.8)	0.28 (0.07-1.07)	0.06	0.24 (0.06-0.98)	0.047
Sep 2013-Aug 2014	3 (3.0)	97 (97.0)	0.21(0.05-0.76)	0.018	0.17 (0.05-0.67)	0.01
Sep 2014-Aug 2015	6 (6.7)	83 (93.3)	0.48 (0.16-1.36)	0.16	0.45 (0.14-1.43)	0.18
Site						
Mekelle	12 (8.6)	127 (91.4)	ref			
Alamata	1 (1.4)	72 (98.6)	0.15 (0.02-1.15)	0.07	0.14 (0.02-1.18)	0.07
Shire	5 (6.8)	68 (93.2)	0.78 (0.26-2.30)	0.65	0.53 (0.16-169)	0.28
Humera	9 (6.6)	128 (93.4)	0.74 (0.3-1.83)	0.52	0.64 (0.23-1.72)	0.38

^a^Patients with both PTB & EPTB were not included; ^b^Analysis limited to the smear-positive cases; ^c^Analysis limited to patients with known HIV status; *HIV* human immunodeficiency virus; *Kg* kilogram; *PTB+* smear-positive pulmonary tuberculosis; *PTB-* smear-negative pulmonary tuberculosis; *EPTB* extra-pulmonary tuberculosis

## Discussion

In this study, the overall treatment success was found to be 94% for the prisoners that complete their treatment while in prison. However, all the prisoners transferred to other prisons or released during their treatment were not appropriately linked to health facilities and might be lost to treatment follow-up. TB treatment category, the year of treatment, and sputum non-conversion at the second-month check-up (for smear-positive cases) were statistically associated with unsuccessful treatment outcome.

The observed treatment success rate (94%) is slightly higher than the findings from similar studies in the general population of Tigray (89.2%) [18], northeast Ethiopia (90.1%) [19] and is in the range of the target level set by End TB Strategy (a TSR of ≥90%) [20]. It also remains remarkably higher than reports from Southern (85.2%) [21], Western (70.8%) [22] and Northwest (85.6%) [23] Ethiopia. More specifically, the loss to follow-up (2.5%) and death rates (1.4%) in our study are lower than the loss to follow-up and death rates recorded in the previous studies [18, 21, 22] which ranged from 3.2 to 35.5% and 3.3 to 58.8%, respectively. In all the above-mentioned studies, the TSR was calculated in a similar way with our study where the transferred out patients were not considered. The discrepancy with findings could partly be attributed to differences in settings that might account for the variation in the DOTs performance. In prisons, it easier to administer DOTs and monitor the progress as the patients are accessible to the prison health personnel [24]. In addition, we observed that the prison health personnel provided the TB drugs under strict supervision not only during the first 2 months but also during the 4 months of the continuation phase, which might result in an increased likelihood of better treatment outcome in prisons.

When compared with prison specific studies, the overall TSR in this study (94%) is higher than the findings from the prison in Gondar which ranged from 42 to 80% across the 10 years [13]. The time difference could not exclusively be the reason for this discrepancy because the TSRs in this study were still smaller than the TSR in our study during the years that overlapped with our study period. Other factors such as differences in the healthcare delivery service between the prisons of the two regions and the commitment of the prison health personnel in delivering the DOTS service might be possible reasons. Higher rates of loss to follow-up were reported elsewhere in prisons in Uganda (43.0%) [10], and Brazil (13.0%) [9]. Similarly, the 1.4% death rate observed in this study is lower than those recorded in Ugandan (5.0%) [10], South African (1.8%) [11] and Brazilian (2.0%) prisons [9]. One possible explanation for this variation could be the difference in the burden of HIV co-infection, which has been shown to be associated with unsuccessful treatment outcome [19, 25]. For example, 54% of the study participants in the South African prison [11] were co-infected with HIV [11] whereas only 11% of the TB cases had HIV co-infection in our study.

Our study demonstrates that the DOTS program is effectively functioning for prisoners that complete their TB treatment while in the study prisons. However, the absence of appropriate linkage for those prisoners transferred to other prisons or released during their treatment raises a public health concern. It is more likely for such patients to end up with a loss to follow-up or treatment failure if not death. Those patients who survived would be at high risk to develop of drug-resistant TB [10]. This also implies that the community would be at risk of being infected with a resistant strain from these patients. For the national TB program to be successful, governmental and non-governmental organizations (NGOs) should take the initiative in establishing improved linkage between the prison and public health facilities. For example, a mobile-based communication system involving the prison health personnel, the health professionals at a public health facility, and the released prisoners could be a good approach in tracking released prisoners and reducing loss to treatment follow-up [26].

In this study, as it could be expected, previously treated cases were more likely to have unsuccessful treatment outcome compared to new cases which is consistent with several previous reports [11, 18]. One of the reasons could be the high level of treatment failure and hence possible development of drug-resistant strains in retreatment cases [27]. In addition, patient-related behavior might also have contributed to the unsuccessful outcome; a study indicated patients that were already lost to follow-up previously could be reluctant and tend to interrupt their treatment again [28]. Due attention should be given to such patients to make sure that they are taking the full course of treatment and immediate referral should be done for MDR screening if they already had a treatment failure. Year of treatment was also associated with variations in treatment outcome; in general, those treated during the earlier year (September 2010–Augst 2011) were more likely to have unsuccessful outcome compared to the latest years, indicating improvements in the DOTS performance over years. This could be due to the increasing efforts of the national TB control program in introducing and implementing the TB/HIV collaborative activities in healthcare settings and prisons in recent years [29].

Unlike several previous reports [11, 19, 25], in this study, HIV co-infection was not associated with unsuccessful outcome. The difference in the sample size and burden of the co-infection rate might be possible reasons for this. In addition, the prison health personnel reported that they routinely supervise the TB treatment progress of prisoners including those co-infected with HIV which might have contributed to the improved outcome in both groups. Though it was not associated with unsuccessful outcome, body weight at initiation of anti-TB treatment (<50 Kgs) was found to be a significant predictor of death of the patients, which is in agreement with previous reports [30, 31]. The relationship between TB and malnutrition is bidirectional. Severe TB disease renders patients to be malnourished and malnourished individuals are at a high risk to develop severe TB diseases and end up with unfavorable treatment outcomes [32]. Hence, the death of patients might be attributed to the severity of the TB disease itself or due to the malnutrition and associated consequences. Closer nutritional monitoring and earlier initiation of nutrition support are important to rescue severely malnourished TB patients [32]. In smear-positive cases, sputum non-conversion at the second-month check-up was a predictor of unsuccessful outcome. This might be partly related to the drug resistance development. In 2015, the incidence of multidrug-resistant/rifampicin-resistant (MDR/RR-TB) in Ethiopia was estimated to be 2.7% among the new and 14% among retreatment TB cases [20]. In the Tigray region of Ethiopia, 55% of the presumptive MDR cases have been shown to harbor MDR strains [33]. We suggest that such prisoners should be immediately referred for DST.

Furthermore, an overview of the TB profile data showed that the majority of the patients in our study were EPTB and PTB− cases, which is consistent with the previous reports from the general population in Ethiopia [34, 35]. The exact causes for the high proportion of EPTB in Ethiopia remain unknown, however, the high potential for a wrong diagnosis (due to the poor diagnostic facility), and poor immunologic and nutritional status have been shown to be associated with high rates of EPTB and PTB− cases [36, 37]. This might suggest the need to incorporate a more accurate diagnostic test, such as the GeneXpert MTB/RIF assay, in Ethiopian prisons.

This study has some limitations mainly inherited from the retrospective design. As we relied on historical records, we were not able to add some important variables such as alcoholism, smoking, substance abuse, nutritional status, and lack of family support, which are known to be related to the variations in the TB treatment outcome [38]. Tracking the prisoners transferred between prisons was also not possible, as the information was not clearly indicated in the treatment recording protocol.

## Conclusions

The mean treatment success rate of the prisoners in the study prisons was quite satisfactory when gauged against the target level set by the End TB Strategy. However, the lack of appropriate linkage and tracking systems for those prisoners transferred or released before their treatment completion would have a negative implication for the national TB control program as such patients might interrupt their treatment and develop drug-resistant TB. Being in a re-treatment regimen and sputum non-conversion at the second-month check-up were significantly associated with unsuccessful outcome among all forms of and smear-positive TB cases, respectively. The concerned authorities should take an urgent action to help establish improved linkages between the prison and public health facilities to prevent possible loss to follow-up of released prisoners and due attention should be given to the previously treated cases.

## Abbreviations

AFB: Acid-fast bacilli
DOTS: Directly observed treatment short-course
DST: Drug susceptibility testing
EPTB: Extra-pulmonary tuberculosis
HIV: Human immunodeficiency virus
MDR-TB: Multidrug-resistant tuberculosis
PTB−: Smear-negative pulmonary tuberculosis
PTB+: Smear-positive pulmonary tuberculosis
TB: Tuberculosis
TSR: Treatment success rate
WHO: World Health Organization
